# Dub3 controls DNA damage signalling by direct deubiquitination of H2AX

**DOI:** 10.1002/1878-0261.12097

**Published:** 2017-06-18

**Authors:** M. Rocío Delgado‐Díaz, Yusé Martín, Anna Berg, Raimundo Freire, Veronique A.J. Smits

Volume 8, Issue 5, pages 884‐893. First published: 18 March 2014.

https://doi.org/10.1016/j.molonc.2014.03.003

The Dub3 siRNA oligonucleotide sequence mentioned in this article is incorrect. The correct sequence of the used Dub3 siRNA oligo is GGAGAUCCAAAGGGAAGAAdTdT.

The authors apologise for any inconvenience caused by this error.

